# Effects of Taxon Sampling in Reconstructions of Intron Evolution

**DOI:** 10.1155/2013/671316

**Published:** 2013-03-03

**Authors:** Mikhail A. Nikitin, Vladimir V. Aleoshin

**Affiliations:** Belozersky Institute for Physicochemical Biology, Lomonosov Moscow State University, Moscow 119991, Russia

## Abstract

Introns comprise a considerable portion of eukaryotic genomes; however, their evolution is understudied. Numerous works of the last years largely disagree on many aspects of intron evolution. Interpretation of these differences is hindered because different algorithms and taxon sampling strategies were used. Here, we present the first attempt of a systematic evaluation of the effects of taxon sampling on popular intron evolution estimation algorithms. Using the “taxon jackknife” method, we compared the effect of taxon sampling on the behavior of intron evolution inferring algorithms. We show that taxon sampling can dramatically affect the inferences and identify conditions where algorithms are prone to systematic errors. Presence or absence of some key species is often more important than the taxon sampling size alone. Criteria of representativeness of the taxonomic sampling for reliable reconstructions are outlined. Presence of the deep-branching species with relatively high intron density is more important than sheer number of species. According to these criteria, currently available genomic databases are representative enough to provide reliable inferences of the intron evolution in animals, land plants, and fungi, but they underrepresent many groups of unicellular eukaryotes, including the well-studied Alveolata.

## 1. Introduction

Introns are noncoding sequences inside many eukaryotic genes. Their abundance may vary several orders of magnitude, from hundreds of thousands in mammalian genomes to less than 100 in the genome of *Saccharomyces cerevisiae*. The origin and evolution of introns is a highly controversial topic despite 25 years of research. The variation of intron content between different lineages suggests a high variation in the rate of intron gain and loss, which may relate to differences in population size, absence or presence of the sexual process, activity of transposable elements, properties of the splicing mechanism, and many other characteristics of genomes, organisms, and populations. 

Intron sequences evolve at a high rate, and the negative selection typically stabilizes only few nucleotide positions in splicing sites and the branch point. Interestingly, a significant portion of introns occupy the same positions in the same genes in species that diverged billions years ago (20% of common introns in mammals and *Arabidopsis thaliana *for the set of 684 conservative genes [1]). These introns were interpreted either as ancestral, originating before the divergence of major eukaryotic lineages, or as convergently inserted in the same positions due to sequence properties (the “protosplice sites”). Both explanations can be true for different subsets of introns in same genome; however, the proportion of ancestral and convergent intron positions in shared introns is controversial. Its estimates vary from 2% to 18% [2] and even more than 50% [3]. The latter estimate was obtained using ad hoc algorithm and datasets and is not directly comparable to others. Functional explanations of the extremely low rate of intron loss are not known. Many introns were found to contain functional elements, such as transcriptional and splicing regulators [4], small regulatory RNAs which produced during the subsequent cleavage of the excised intron, nonsense-mediated decay signals, signals of nuclear export, and others. However, these processes are not evolutionarily conserved, which therefore does not explain the survival of introns for billions of years.

With the influx of new genomic data, our view of intron evolution changes. For example, the high intron content in vertebrate genomes was initially interpreted as a derived feature of this lineage. However, genomic analysis of the polychaete *Platynereis dumerili* [5] suggested that most vertebrate introns were already present in ancestral Bilateria and subsequently lost in insect and nematode lineages. In next years, analyses of genomic sequences of cnidarians [6], Placozoa [7], sponges [8], choanoflagellate [9], and early-diverging fungi [10] pushed the origin of abundant vertebrate introns back to the ancestral metazoans and, for some, even earlier, to the unicellular common ancestor of animals and fungi. A recent study of the intron evolution in Alveolata and stramenopiles with data on 23 species infers a highly intron-rich ancestors of Alveolata and Alveolata+stramenopiles, with latter containing more introns per gene as humans [11]. This is unexpected, because all extant members of these groups exhibit a low or at best moderate (*Thalassiosira pseudonana*) intron density [12]. These examples raise the following questions.

- How does the available taxon sampling affect our studies of the evolution of introns?
- How can the taxon sampling be tested to provide accurate reconstructions?
- Which species should be added to compensate for an incomplete taxon sampling?

## 2. Materials and Methods

**Figure 1:**
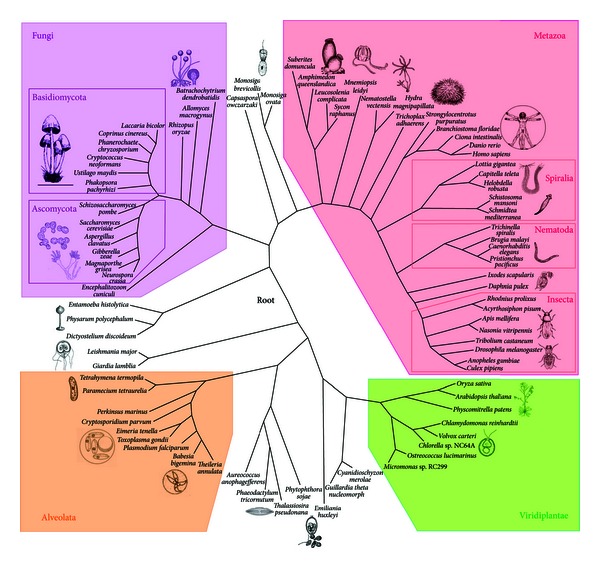
The phylogenetic tree for the initial species set according to [13–20].

To address these questions, we compiled dataset of intron-exon structures of two ribosomal protein genes (rpS5 and rpL12) for 80 species representing three major eukaryotic groups, Opisthokonta, Plantae, and SAR (Stramenopiles-Alveolata-Rhizaria), using data from publicly available databases of completed and ongoing genome projects. Phylogenetic relations of the analyzed species according to recent studies [13–20] are depicted on Figure 1. For unannotated data, putative rpS5 and rpL12 cDNA and genomic sequences were found with BLAST, and intron-exon boundaries were established using Genscan [21]. We generated 660 random subsamplings ranging from 15 to 75 species from the initial 80 species set using custom Python scripts (100 subsamplings with 15 and 20 species, 80 with 25, 60 with 30 and 35 each, 40 with 40, 45, 50 and 55 each, 30 with 60 and 65, and 20 with 70 and 75 species). The Csuros [22] and NYK [2] algorithms of inferring intron evolution were run on each of these subsamplings.

Results were imported in STATISTICA 8 for statistical analysis and scatterplot generation. If no members of a taxon were present in a subsampling, this subsampling was discarded from calculations and scatterplots for this taxon. 

## 3. Results

### 3.1. Overview

**Figure 2:**
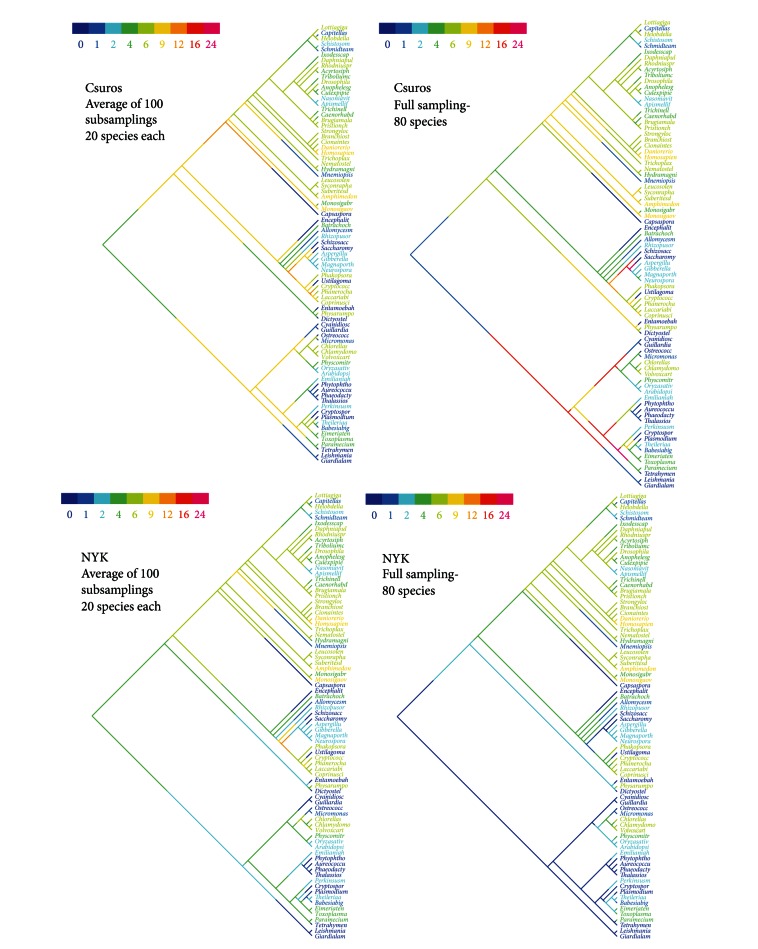
Examples of the intron evolution reconstructed with different taxon samplig size. The intron density on each branch (in introns/kb) is color-coded according to scale in the upper left corner. Upper row: Csuros algorithm, lower row: NYK algorithm. Left column: averaged intron densities using 100 subsets of 20 species each. Right column: full set of 80 species.

We reconstructed intron phylogenies for the full set of 80 species and for 660 random subsamplings using the algorithms by Csuros and NYK. As depicted in Figure 2, different taxon samplings produce different results. In many smaller subsets, there were no members of a particular taxa. These subsets were excluded from calculations of average ancestral intron densities for these taxa. For example, in 105 out of 660 subsets, there were no nematodes, and they were excluded from calculations of average intron density in the ancestor of Nematoda.

For the NYK algorithm, the most striking difference is observed in the internal nodes of the bikont half of the eukaryotic tree. Using subsets of 20 species, one can see a more or less constant intron density among the internal branches in different bikont groups such as Alveolata, stramenopiles, and Viridiplantae. The analysis of original set of 80 species inferred almost intronless ancestors for these groups and recent episodes of intron gain along terminal branches. A similar, but less pronounced, pattern is also observed for the Ascomycota, Basidiomycota, and the animal-fungal ancestor. These internal nodes also appear more intron rich when sparse taxon coverage is used. Among the Metazoa and their closest relatives, Choanoflagellata, the results do not change significantly varying the taxon coverage. 

For the Csuros algorithm, significant differences were also observed between broader and narrower taxon samplings. Again, these differences are most prominent on internal branches of the Bikonta and Fungi. For small species sets, the output of Csuros algorithm is similar to that of NYK. Analysis of the complete taxon set of 80 species returns very high intron densities for the ancestors of Sporozoa, Apicomplexa, Alveolata, and Ascomycota, far exceeding the observations in recent organisms. Particularly, in the ancestor of Apicomplexa, the estimated intron density in analysis of 80 taxa equals 22/kb, which is three times higher than in mammals (7/kb).

### 3.2. Specific Effects of Taxon Sampling on Different Nodes

**Figure 3:**
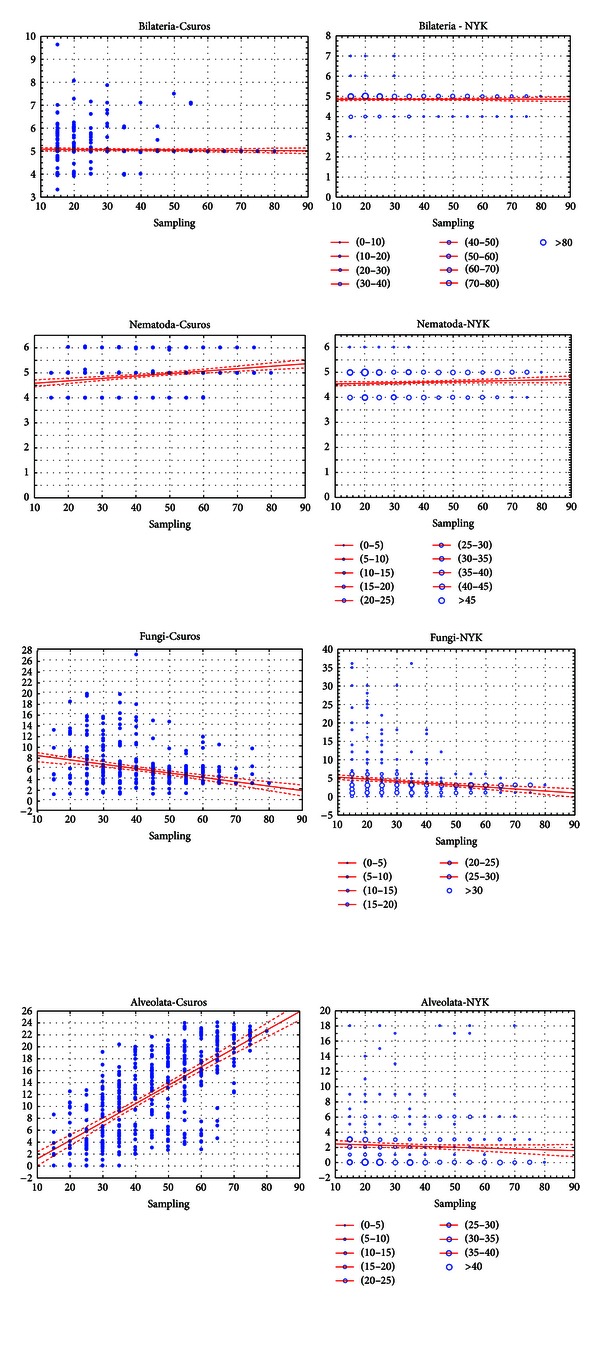
Examples of sampling size-intron density correlation patterns inferred by the Csuros (left) and NYK (right) algorithms. Horizontal axis: number of species in the sampling. Vertical axis: inferred intron density at the node (introns/kb). Note that NYK returns estimations of ancestral intron count rounded down to integers, therefore bubble diagram was used. Bubble size represents number of data points at the same scatterplot coordinates.

As can be noticed, varying the taxon sampling size affects particular nodes (such as Alveolata and Viridiplantae) more than others (e.g., Metazoa).  Figure 3 reproduces this pattern in more detail. For the Bilateria, one can see that average statistics are the same for both algorithms and do not correlate with sampling size. The only observed effect of sampling is a significant dispersion of estimates between smaller taxon sets and uniform patterns when the sampling size is 40 or more species. At internal nodes of Bilateria, such as Nematoda, Insecta, and Spiralia, a significant positive correlation is observed between the intron number and taxon sampling size. Dispersion between samplings of same size is large for smaller samplings and decreases on larger taxon sets. For the Fungi, Basidiomycota, Opisthokonta, and Viridiplantae, a high dispersion is observed for all sampling sizes, albeit less on the larger ones. The average intron density in Fungi and Opisthokonta shows a negative correlation with the sampling size for the both NYK and Csuros algorithms (Table 1), while for the Basidiomycota and Viridiplantae, these correlations are insignificant. The Alveolata exhibit a high correlation of the inferred intron density with the sampling size, however correlation patters are different for the Csuros and NYK algorithms. The two algorithms behave similarly with smaller samplings; however, with larger sets, Csuros infers extremely high intron numbers, and NYK infers their complete absence. Similar patterns were also observed for the common ancestors of Apicomplexa, Sporozoa, and Ascomycota.

### 3.3. Factors Affecting the Reconstruction

One may discuss three factors that influence ancestral reconstructions under varying the taxon sampling. First, broader sampling usually produces more descendants of a given internal node in analyses. Second, the number of outgroup taxa also depends on the sampling size. Third, particular key taxa may strongly affect reconstruction when present in the dataset, which are more likely be found in larger samplings. These three factors may contribute differently and produce a mixed effect. To evaluate their contributions separately, we performed a multiple regression analysis using the numbers of descendants and outgroup species and the presence/absence of particular descendants as independent variables. The results are presented in Table 1.

**Figure 4:**
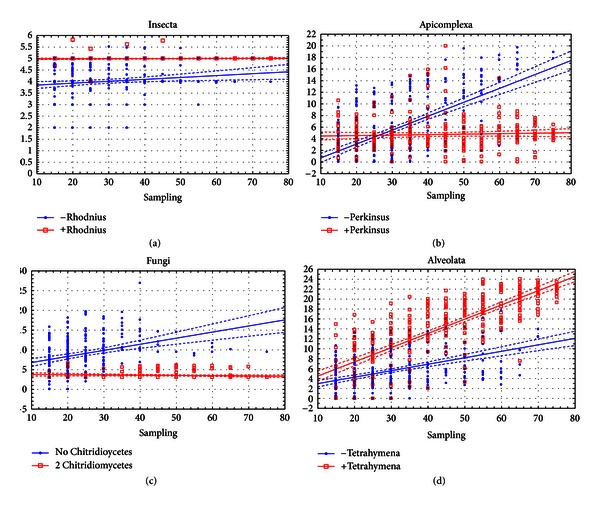
Effects of critical species sampling on the behavior of the Csuros algorithm. Horizontal axis: number of species in the sampling. Vertical axis: inferred intron density at the node (introns/kb).

**Figure 5:**
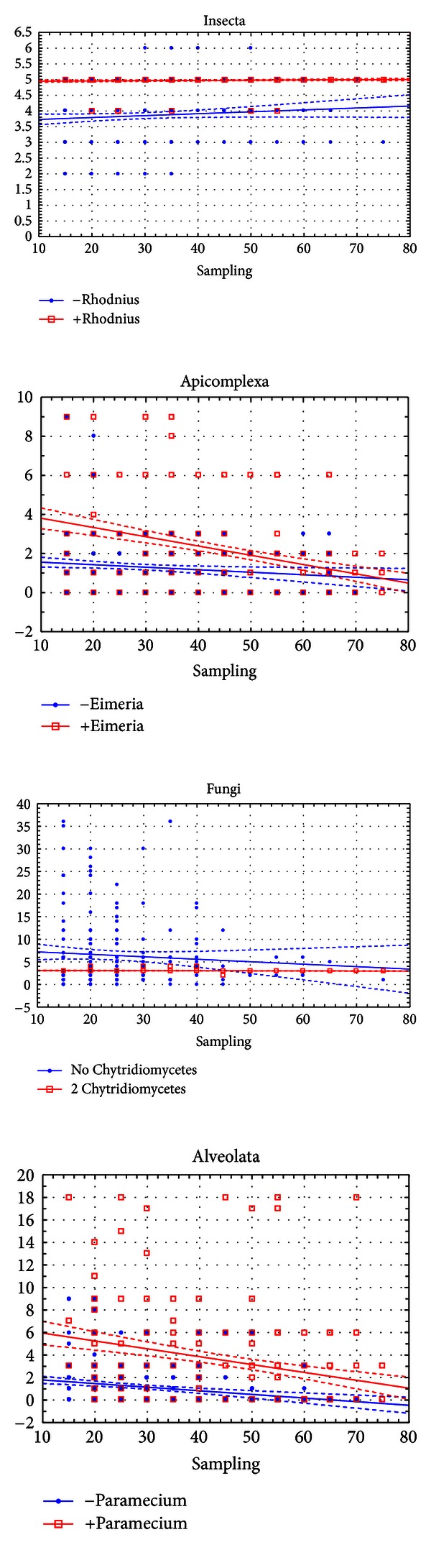
Effects of critical species sampling on the behavior of the NYK algorithm. Horizontal axis: number of species in the sampling. Vertical axis: inferred intron density at the node (introns/kb).

The regression analysis shows that for the Insecta and Spiralia, the main affecting factor is the presence of particular species in the dataset for both Csuros and NYK algorithms. These species are *Rhodnius prolixus* for the Insecta and *Lottia gigantea* for the Spiralia (partial correlation coefficients beta equal 0.65 and 0.67). Correlations with the number of descendants for these nodes range within 0.35–0.37 for Csuros and 0.12–0.30 for NYK. Correlations with the sampling size are less pronounced. For Insecta, we also found that correlation of results with the sampling size is significant only when *Rhodnius prolixus* is not sampled (Figures 4 and 5).

For Nematoda, presence of each of the four of its descendants shows a significant effect, with beta positive, ranging within 0.33–0.39 for *Trichinella spiralis*, *Brugia malayi,* and *Pristionchus pacificus,* and negative −0.20 for *Caenorhabditis elegans.* These correlations are almost the same for Csuros and NYK algorithms.

For other nodes, significance of different factors depends on the algorithm choice.

*When the Csuros algorithm is used,* for the nodes of Fungi, Basidiomycota, Apicomplexa, and Viridiplantae, the presence of some species affects inferences of intron evolution more than other factors. These species are *Allomyces macrogynus* and *Batrachochytrium dendrobatidis* for the Fungi (beta = −0.48 and −0.53), *Phakopsora pachyrhizi* for the Basidiomycota (beta = −0.32), *Chlorella variabilis* for the Viridiplantae (beta = −0.23), and *Perkinsus marinus* for the Apicomplexa (beta = −0.26). Furthermore, a significant correlation of the ancestral intron density with the sampling size and the number of descendants is observed only when the critical species are absent (both species absent in case of Fungi).

For the Ascomycota, Alveolata, and Sporozoa, critical species are not easily identified. Presence of every descendant of these nodes shows a significant correlation with the inferred ancestral intron density. Still, some species are more important than others. Among Ascomycota, these are yeasts *Saccharomyces cerevisiae* and *Schizosaccharomyces pombe *(beta = 0.46 and 0.41, while for other species is lower than 0.20). For the Sporozoa, most variation is due to *Cryptosporidium parvum* (beta = 0.45, for others less then 0.23), and in the Alveolata, these are two ciliates *Paramecium tetraurelia* and *Tetrahymena thermophila* (beta = 0.35 and 0.61, while lower than 0.15 in other cases). Unlike the aforementioned nodes, for the Ascomycota, Sporozoa, and Alveolata, most significant correlation of the ancestral intron density with sampling size and descendants number is observed only when critical species are present in subsets. 

*For NYK algorithm,* certain species usually show the highest impact on reconstructions of ancestral introns, but the overall picture is often more complicated. For the Fungi, similarly to the Csuros algorithm, the critical species are *Allomyces macrogynus*, *Batrachochytrium dendrobatidis,* and *Encephalitozoon cuniculi.* Presence of any of them greatly reduces the dispersion between subsamplings and prevents very high or very low estimates. For the Alveolata, the critical species are again ciliates, but presence of *Paramecium tetraurelia* positively correlated with the ancestral intron density, and that of *Tetrahymena thermophila*—negatively. In the Sporozoa, the highest correlation is observed for *Cryptosporidium parvum* and *Eimeria tenella*, again with opposite signs. In the Apicomplexa, there are four species that exhibit significant effects—*Cryptosporidium parvum, Eimeria tenella*, *Paramecuim tetraurelia, *and *Tetrahymena thermophila. *Among the Viridiplantae, there are two important descendants, *Ostreococcus tauri* and *Oryza sativa*, and two important outgroup species, *Paramecuim tetraurelia *and *Tetrahymena thermophila. *A significant effect on the variations of ancestral intron count for the Basidiomycota was found for six species: four descendants (*Cryptococcus neoformans, Phakopsora pachyrhizi, Ustilago maydis,* and *Coprinus cinereus*) and two outgroup species (*Allomyces macrogynus* and *Batrachochytrium dendrobatidis*). Analyses for the Ascomycota robustly produce the estimation of 2 introns/kb, with only 13 out of 660 subsamplings exhibiting much higher estimates.

One can see that many key species are the same for both algorithms—for Fungi or ciliates for Alveolata. However, there are significant differences in the importance of outgroup species. They are often important for the NYK algorithms but show only minor effects for the Csuros.

## 4. Discussion

### 4.1. Cases of Overestimation of the Ancestral Intron Count

The Csuros algorithm in many cases outputs unrealistic, very high intron densities of 15–20/kb, which is three times higher than the observed values in any recent organism and seems unlikely if we consider the spliceosome positioning on pre-mRNA. Such overestimation is commonly found for the Alveolata, Sporozoa, and Ascomycota and also occurs in a portion of subsamplings for the Apicomplexa, Fungi, and Basidiomycota. Our analysis of these anomalies shows that they occur when very intron-poor taxa occupy the basal position among descendants of a node. Yeasts, ciliates, and *Cryptosporidium parvum* are intron-poor and basal for the Ascomycota, Alveolata, and Sporozoa, respectively, in our full set of 80 species. The chance that these species are present in the analysis increases with the subset size, leading to a high positive correlation of the inferred intron density with sampling size. With the Fungi, Basidiomycota, and Apicomplexa, the full set contains relatively intron-rich basal species (*Allomyces macrogynus*, *Batrachochytrium dendrobatidis*, *Phakopsora pachyrhizi,* and *Perkinsus marinus*), followed by intron-poor branches (yeasts, *Ustilago maydis*, and *Cryptosporidium parvum*). For these nodes, the inferred ancestral intron densities show a bimodal distribution, depending on which species happens to be basal in subsamplings. Interestingly, even for the Metazoa, there are several cases when the Csuros algorithm overestimates the ancestral intron density to exceed 10/kb. In all such cases, we found that the extremely intron-poor ctenophore *Mnemiopsis leydi* in these subsamplings falls in the basal position within Metazoa, while all intron-rich poriferan species are absent.

The NYK algorithm is also prone to overestimation of the ancestral intron count. This is often observed with the Fungi and sometimes with the Basidiomycota, Ascomycota, and Alveolata. As we have found, the prerequisite for such an overestimation is the absence of *Allomyces macrogynus* and *Batrachochytrium dendrobatidis* (for Fungi, Basidiomycota, and Ascomycota), presence of *Saccharomyces cerevisiae* and *Schizosaccharomyces pombe *(for Ascomycota only), and presence of *Paramecuim tetraurelia* and *Tetrahymena thermophila* (for Alveolata). 

These conditions are similar for those for the Csuros algorithm, but NYK shows a much lesser degree of systematic overestimation. It is especially shown with the example of Ascomycota; the overestimation by Csuros was found in more than half of subsamplings, while by NYK—only in 13 out of 660 subsamplings. The factor analysis also shows that the set of outgroup taxa does not affect the reconstructions with the Csuros algorithm but is important in the case of NYK. 

### 4.2. How Many Taxa Are Enough?

**Figure 6:**
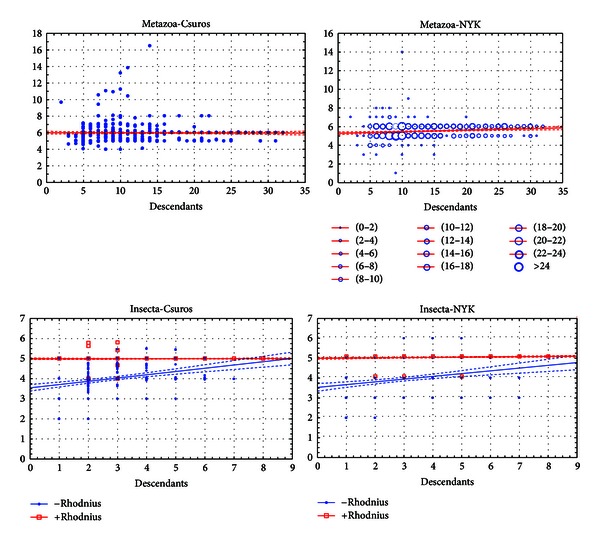
Effects of the number of descendants and sufficiency of taxon sampling. Horizontal axis: number of species in the sampling. Vertical axis: inferred intron density at the node (introns/kb).

Using the nodes where different algorithms produce similar results, we could evaluate the number of descendants required for accurate reconstructions of intron evolution. In the Spiralia and Insecta, basal intron-rich species (*Lottia gigantea* and *Rhodnius prolixus*) strongly affect the results, while in the case of 8-species sets, the results with and without *Rhodnius prolixus* are very similar (Figure 6). For the Spiralia, a similar trend exists, however, less pronounced due to only 5 available descendants. For the Bilateria and Metazoa, reconstructions are the same with the both algorithms and almost do not depend on sampling, possibly due to a high number of descendants in the sampling (24 and 32, resp.). The average intron count for these nodes does not correlate with the sampling size even if all subsamplings with more than 10 descendants of these nodes are discarded. So, the Metazoa and Bilateria are not very useful for estimating the sampling adequacy. With the results for Insecta, we conclude that 8–10 species should be enough given no catastrophic intron loss among the descendants of the analyzed node. The results obtained for the Bilateria and Metazoa do not contradict with this conclusion.

### 4.3. Comparison with Earlier Intron Evolution Studies

The recent work by Csuros et al. [23] uses an MCMC-based algorithm for the reconstruction of the intron evolution and a broad sampling of 99 species. It also shows that the reliability of the reconstruction of the intron evolution differs between nodes. Their algorithm produced not only inferred estimates of the ancestral intron density, but also its Bayesian posterior distributions. Similarly to our results, the estimates for the Metazoa and Bilateria are robust, the Alveolata exhibit a significant uncertainty, and the stramenopiles-Alveolata (SAR) group or Amoebozoa posterior distribution shows that the estimations are unreliable. Despite the broad taxon sampling, the authors do not use data on such deep-branching intron-rich species as *Perkinsus marinus* in the Alveolata and *Physarum polycephalum* in the Amoebozoa. We predict that adding these species to the authors' 99-species set will stabilize the results for the Alveolata and Amoebozoa, respectively. 

The Csuros's algorithm tested in our work was used, for example, in the study [11] of intron evolution in the Alveolata and stramenopiles. The authors report unusually high estimates of the ancestral intron densities for many nodes. The highest was 7.5 introns/kb in ancestor of Alveolata, which is 20% higher than in the most intron-rich modern organisms. Our observations suggest that this is likely a systematic bias of the Csuros algorithm. This view is supported by the results obtained with the MCMC algorithm from [14], where the inferred intron density in the ancestral Alveolata is more conservatively estimated at 5.0 introns/kb.

It is of interest to compare the results of our study with [10]. Stajich et al. studied the intron evolution in Fungi, using a sampling of 25 species and four algorithms, NYK, Csuros, Roy-Gilbert, and EREM. The results of all algorithms were in good agreement for most nodes, including the Ascomycota. No systematic overestimations by any algorithm were detected, unlike our work and [11]. There might be two reasonable explanations: (1) a broad gene sampling (1161) allowed to correctly estimate the rate of intron loss even under the low intron density in the basal ascomycete *Schizosaccharomyces pombe*; (2) a broad species sampling (5) of extremely intron-poor hemiascomycete yeasts and a differential intron loss in them allowed for the conservation of a considerable subset of the ancestral introns. Unfortunately, a broader gene sampling is not always available for groups with an extensive gene loss. For example, for 23 species in [11] (11 stramenopiles and Alveolata and 12 outgroup species), only 394 orthologous genes were present in at least 18 out of 23 taxa. With methods that do not allow for missing data, like NYK, gene sampling would be even poorer. 

## 5. Conclusions

We observe that the number and composition of taxa often have a strong impact in reconstructions of the intron evolution. While insignificant for some nodes, such as the Bilateria and Metazoa in our analyses, it can be significant for many others. A stronger influence of taxon sampling is observed in nodes with descendants possessing an intensive intron loss. If such a descendant occupies the basal position, the ancestral intron reconstructions are often unreliable. In the indicated cases, the Csuros algorithm exhibits a trend to systematically overestimate the ancestral intron count. Overestimations also occur with the NYK algorithm, however, under a more complex set of conditions and in our analyses were frequently observed only in the node of Fungi. If a group suffers from massive intron loss, a recommended strategy to improve the accuracy of inferring the intron evolution is to identify and add to dataset a deep-branching member of this group with a high intron density, such as *Perkinsus marinus* in the Apicomplexa. 

## Figures and Tables

**Table 1:** Partial correlations of the sampling size, number of descendants, outgroups, and presence/absence of key species with intron densities in ancestral nodes. Italics are correlations significant at *P* level > 0.05. In **bold** are highest correlations for the node.

NYK	Sampling		Descendants		Outgroup-partial		1st descendant			2nd descendant		
	Beta	*P* level	Beta	*P* level	Beta	*P* level	Beta	*P* level	Name	Beta	*P* level	Name
Fungi	*−0.24 *	*0.00 *	*−0.09 *	*0.02 *	−0.01	0.72	**−0.27**	**0.00**	**Allomyces**	*−0.18 *	*0.00 *	*Encephalitozoon *
Bilateria	*0.16 *	*0.00 *	0.05	0.21	−0.04	0.26	**−0.25**	**0.00**	**Trichinella**	*0.22 *	*0.00 *	*Daphnia *
Spiralia	*0.14 *	*0.01 *	*0.30 *	*0.00 *	−0.08	0.05	**0.68**	**0.00**	**Lottia**	*0.30 *	*0.00 *	*Helobdella *
Nematoda	*0.20 *	*0.00 *	*0.21 *	*0.00 *	−0.08	0.05	**−0.29**	**0.00**	**Caenorhabditis**	*0.25 *	*0.00 *	*Brugia *
Insecta	*0.33 *	*0.00 *	*0.35 *	*0.00 *	−0.08	0.05	**0.66**	**0.00**	**Rhodnius**	*0.24 *	*0.00 *	*Drosophila *
Ascomycota	−0.09	0.07	0.07	0.09	*−0.09 *	*0.03 *	**−0.13**	**0.01**	**Schizosacch**	*0.10 *	*0.03 *	*Magnaporthe *
Basidiomycota	**−0.31**	**0.00**	*−0.13 *	*0.00 *	*−0.09 *	*0.02 *	*−0.22 *	*0.00 *	*Cryptococcus *	*−0.15 *	*0.00 *	*Ustilago *
Viridiplantae	−0.06	0.24	0.04	0.29	*−0.09 *	*0.03 *	**−0.24**	**0.00**	**Ostreococcus**	−0.17	*0.00 *	*Oryza *
Alveolata	−0.09	0.09	*0.13 *	*0.00 *	*−0.15 *	*0.00 *	**0.45**	**0.00**	**Paramecium**	−0.23	*0.00 *	*Tetrahymena *
Apicomplexa	*−0.19 *	*0.00 *	*0.10 *	*0.02 *	*−0.17 *	*0.00 *	**0.35**	**0.00**	**Eimeria**	−0.24	*0.00 *	*Cryptosporidium *
Sporozoa	*−0.12 *	*0.02 *	0.02	0.60	−0.07	0.08	**0.30**	**0.00**	**Eimeria**	**−0.30**	**0.00**	**Cryptosporidium**

Csuros	Sampling		Descendants-partial		Outgroup		1st descendant			2nd descendant		
	Beta	*P* level	Beta	*P* level	Beta	*P* level	Beta	*P* level	Name	Beta	*P* level	Name

Fungi	*−0.34 *	*0.00 *	*−0.24 *	*0.00 *	0.02	0.63	**−0.53**	**0.00**	**Batrachochytrium**	*−0.48 *	*0.00 *	*Allomyces *
Bilateria	−0.05	0.30	−0.09	0.09	0.02	0.65	**−0.15**	**0.00**	**Nematostella**	*−0.13 *	*0.00 *	*Branchiostoma *
Spiralia	*0.13 *	*0.01 *	*0.36 *	*0.00 *	*−0.10 *	*0.01 *	**0.71**	**0.00**	**Lottia**	*0.48 *	*0.00 *	*Helobdella *
Nematoda	*0.26 *	*0.00 *	**0.36**	**0.00**	−0.06	0.19	*0.39 *	*0.00 *	*Brugia *	*0.34 *	*0.00 *	*Trichinella *
Insecta	*0.34 *	*0.00 *	*0.36 *	*0.00 *	*−0.08 *	*0.04 *	**0.64**	**0.00**	**Rhodnius**	*0.30 *	*0.00 *	*Drosophila *
Ascomycota	**0.61**	**0.00**	*0.54 *	*0.00 *	*0.23 *	*0.00 *	*0.46 *	*0.00 *	*Saccharomyces *	*0.41 *	*0.00 *	*Schizosaccharomyces *
Basidiomycota	0.09	0.06	0.07	0.09	0.07	0.08	**−0.32**	**0.00**	**Phakopsora**	*0.19 *	*0.00 *	*Cryptococcus *
Viridiplantae	−0.03	0.51	0.00	0.92	0.06	0.17	**−0.23**	**0.00**	**Chlorella**	*0.12 *	*0.00 *	*Ostreococcus *
Alveolata	**0.71**	**0.00**	*0.52 *	*0.00 *	*0.18 *	*0.00 *	*0.61 *	*0.00 *	*Tetrahymena *	*0.35 *	*0.00 *	*Paramecium *
Apicomplexa	*0.20 *	*0.00 *	*0.12 *	*0.00 *	*0.09 *	*0.02 *	**−0.26**	**0.00**	**Perkinsus**	*0.14 *	*0.00 *	*Cryptosporidium *
Sporozoa	*0.45 *	*0.00 *	*0.47 *	*0.00 *	0.00	0.92	**0.49**	**0.00**	**Cryptosporidium**	*0.28 *	*0.00 *	*Toxoplasma *
